# Isolated Left Peroneal Neuropathy Following Total Laparoscopic Hysterectomy: A Case Report

**DOI:** 10.7759/cureus.103226

**Published:** 2026-02-08

**Authors:** Hector A Hurtado Bravo, Luz Maria Bravo Rodriguez

**Affiliations:** 1 Faculty of Medicine, University La Salle México, Mexico City, MEX; 2 Gynecological Endoscopic Surgery, Hospital Regional Adolfo López Mateos, Institute for Social Security and Services for State Workers (ISSSTE), Mexico City, MEX

**Keywords:** deep peroneal nerve, motor neuropathy, nerve compression, steppage gait, total laparoscopic hysterectomy

## Abstract

Neuropathy of the lower extremities is a rare surgical complication, usually caused by compression, stretching, or direct injury to the nerve during the procedure. Although such injuries have been documented in relation to laparotomies, there are few reports concerning minimally invasive procedures. Here, we present the case of a 46-year-old woman with a long-standing history of abnormal uterine bleeding. Imaging studies revealed large uterine myomatosis, indicating the need for a total laparoscopic hysterectomy. During the immediate postoperative period, the patient developed a steppage gait and an inability to dorsiflex her left foot, which is consistent with peroneal nerve neuropathy. The clinical course and additional tests confirmed the diagnosis, and the patient made a progressive functional recovery following rehabilitation. This case highlights the importance of proper surgical positioning and ergonomic measures during laparoscopic surgery to prevent compressive nerve injuries, as well as the importance of early identification to promote favorable clinical outcomes.

## Introduction

Motor neuropathy, also known as neuropathic compression syndrome, is a group of neurological symptoms that result from the compression or damage to a peripheral nerve in its anatomical pathway. This compression usually occurs at the level of osteofibrous passages or due to prolonged extrinsic compression. The main pathophysiological mechanisms are direct compression, excessive stretching, and neural ischemia, which can occur in traumatic or tumor contexts or in association with positions maintained during surgical procedures [1].

The common peroneal nerve, a lateral branch of the sciatic nerve, is particularly susceptible to this type of injury due to its superficial course around the head of the fibula and the lack of supporting tissue that protects it from external forces. After surrounding the neck of the fibula, the nerve anatomically divides into superficial and deep branches. These are responsible for eversion and dorsiflexion of the foot, respectively. This explains the significant functional impact of injury to the nerve [2].

From a clinical point of view, peroneal neuropathy can manifest as pain localized in the region of the fibula neck, paresthesia in the dorsum of the foot, weakness in dorsiflexion and eversion, and, in more severe cases, foot drop with a steppage gait pattern. Electrophysiological tests, particularly electromyography and nerve conduction studies, are essential diagnostic tools for confirming the level and type of nerve injury [2,3].

In the surgical context, nerve injuries of the lower extremities associated with laparoscopic procedures are rare but potentially preventable. It has been reported that patient positioning, especially in prolonged lithotomy and Trendelenburg positions, can cause stretching or compression of peripheral nerves, including the common peroneal nerve, with the risk increasing as the duration of the procedure and pressure on bony prominences increase [4]. Despite this, reports of isolated peroneal neuropathy following total laparoscopic hysterectomy are exceptional.

## Case presentation

A 46-year-old female patient came to the clinic with a history of long-standing abnormal uterine bleeding, characterized by a progressive increase in quantity and duration, with regular menstrual cycles, associated with an increase in abdominal circumference and increased urinary frequency. She reported no other relevant medical history.

Physical examination revealed a globular abdomen due to adipose tissue, with a palpable uterus up to the level of the umbilical scar. Vaginal examination revealed an enlarged uterus, with the fundus occupied by tumors consistent with uterine myomatosis, estimated to be approximately 20 cm in size on bimanual palpation, with no evidence of active bleeding or other relevant pathological findings.

**Figure 1:**
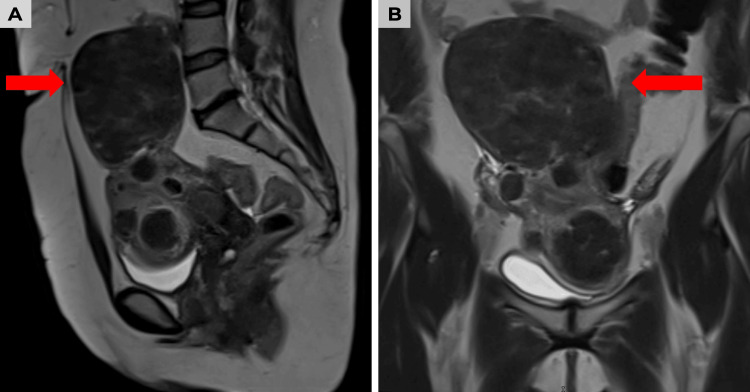
Abdominal-pelvic magnetic resonance imaging in T2 sequence showing an enlarged uterus with dominant myomatous lesion (arrows) (A) Sagittal section with a large myoma dependent on the uterine fundus. (B) Coronal section confirming the dominant lesion with an intramural/subserosal component and mass effect on pelvic structures.

Simple and contrast-enhanced abdominal-pelvic magnetic resonance imaging showed an anteflexed uterus, centered in the midline, measuring approximately 15.7×8.6×13.5 cm, with multiple rounded hypointense lesions on T2 sequences, located in the myometrium and subserosa, with more than 50% intramural component. A pedunculated myoma was identified at the uterine fundus, with the largest lesion measuring approximately 13.5×9×9.7 cm. The endometrium was 3.8 mm thick with no evidence of occupation, the cervix showed Nabothian cysts, and both ovaries were found to be normal. Based on these findings, a diagnosis of FIGO (International Federation of Gynecology and Obstetrics/Fédération Internationale de Gynécologie et d'Obstétrique) type 2, 4, 6, and 7 uterine myomatosis was made [5] (Figure 1A-1B). 

**Figure 2:**
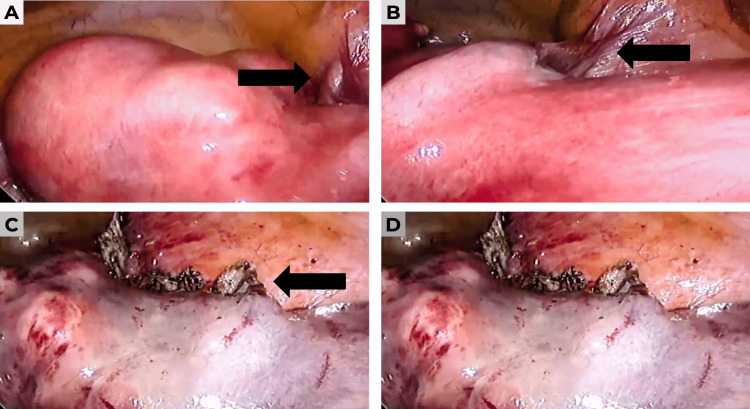
Laparoscopic images showing an enlarged uterus due to large myomatosis (A) Laparoscopic view of the globular uterus with a large subserosal fibroid dependent on the uterine fundus (arrow). (B) Detail of the subserosal fibroid prior to dissection (arrow). (C) Uterus after uterine dearterialization. (D) Subserosal fibroid decapsulated during the laparoscopic procedure.

Preoperative laboratory tests were within normal parameters. Following the corresponding preoperative assessment, the patient was scheduled for a total laparoscopic hysterectomy. During the procedure, a globular uterus measuring approximately 22×15×15 cm was identified, with multiple fibroids, notably a large subserosal lesion dependent on the uterine fundus. The adnexa were observed macroscopically without alterations, and the procedure was completed without apparent complications (Figure 2A-2D). 

The immediate postoperative period passed without surgical complications. However, when she began sitting up and walking, the patient reported paresthesia in the dorsum of her left foot, accompanied by an inability to dorsiflex her ankle. Her gait showed exaggerated elevation of the left knee with initial support on the tip of the foot, consistent with steppage gait.

During her hospitalization, the physical medicine and rehabilitation service confirmed a distal motor deficit in the left pelvic limb, with limited ankle dorsiflexion and superficial sensory disturbances in the areas innervated by the superficial and deep peroneal nerves. Neurotension maneuvers were negative, with no clinical evidence of radicular involvement.

Given the suspicion of peripheral neuropathy, nerve conduction studies and electromyography were performed. The findings were consistent with a predominantly demyelinating motor neuropathy of the left deep peroneal nerve at the proximal level, associated with changes compatible with secondary axonal damage in the tibialis anterior muscle, with involvement at the suprapopliteal and infrapopliteal levels. Based on these results, a diagnosis of moderate neuromusculoskeletal deficiency of the left pelvic limb secondary to common peroneal nerve neuropathy was established (Table 1). 

**Table 1:** Clinical summary of electrophysiological findings

Evaluated structure	Side	Main finding	Clinical interpretation
Common peroneal nerve	Left	Reduced nerve conduction velocity	Predominantly demyelinating neuropathy
Deep peroneal nerve	Left	Reduced motor potential amplitude	Associated axonal damage
Tibialis anterior muscle	Left	Denervation activity and reduced recruitment	Distal motor involvement
Superficial peroneal nerve	Left	Superficial sensory abnormalities	Associated sensory involvement
Evaluated nerves	Right	Parameters within normal limits	Internal control

Following diagnosis, the patient underwent rehabilitation, with progressive recovery of distal motor function in the left pelvic limb and sustained clinical improvement.

## Discussion

Peroneal nerve neuropathy is one of the most common mononeuropathies of the lower extremity and is classically associated with compression, stretching, or ischemia mechanisms, particularly at the head of the fibula, where the nerve is located superficially and has little epineural support, making it highly vulnerable to external factors [6,7]. In the perioperative setting, these injuries are often related to prolonged surgical positioning, especially in positions that involve sustained knee flexion, hip abduction, or direct pressure on bony prominences.

Several studies have indicated that prolonged compression is the main pathophysiological mechanism of peroneal neuropathy, even more so than direct or traumatic injury [8]. This mechanism is particularly relevant in laparoscopic surgical procedures, where the lithotomy position or variants thereof may be maintained for prolonged periods, with decreased sensory perception secondary to anesthesia and absence of spontaneous postural changes by the patient.

The available evidence suggests that most positioning-related peroneal neuropathies correspond to demyelinating lesions, with or without secondary axonal damage, which explains a generally favorable prognosis when the compressive factor is identified and corrected early [6,9]. In the case presented, electromyographic findings demonstrated a predominantly demyelinating pattern with secondary axonal involvement, consistent with a subacute-onset compression neuropathy.

From a diagnostic standpoint, electromyography and nerve conduction studies remain essential tools for confirming the site, severity, and type of nerve injury, as well as for establishing a functional prognosis [6,10]. The identification of decreased conduction velocity, increased latencies, and reduced amplitudes allows differentiation between neuropraxia, axonotmesis, and mixed lesions, as observed in this patient.

With regard to prognosis, multiple reviews agree that compressive peroneal neuropathies show partial or complete recovery in most cases, especially when management is conservative and includes early rehabilitation aimed at preserving range of motion, muscle strength, and gait pattern [11,12]. The favorable outcome observed in this patient, with progressive functional recovery after follow-up with physical medicine and rehabilitation, is consistent with what is described in the literature.

A relevant aspect is the potentially preventable nature of this type of injury. Several authors emphasize that the correct identification of risk areas, the proper use of padding, the limitation of time in risk positions, and the periodic reevaluation of positioning during prolonged procedures are key measures to reduce the incidence of perioperative peripheral neuropathies [8,13]. Although much of the literature has focused on obstetric or non-gynecological surgical contexts, the principles of prevention can be extrapolated to gynecological laparoscopic surgery.

Finally, this case provides relevant clinical evidence by documenting isolated peroneal neuropathy following total laparoscopic hysterectomy, a complication rarely reported in the literature, and reinforces the importance of maintaining a high index of suspicion when gait disturbances or neurological deficits appear in the immediate postoperative period. Early detection allows for timely intervention, prevents prolonged functional deterioration, and improves neuromusculoskeletal outcomes.

## Conclusions

This case highlights the importance of proper patient positioning during laparoscopic surgery as a key measure to prevent not only joint injuries but also peripheral neurological complications. Recognition of anatomical risk areas and pressure points susceptible to nerve compression is essential to minimize adverse events not directly related to the surgical technique. Likewise, early identification of neurological signs in the postoperative period should alert the clinician and prompt a timely evaluation with complementary studies, allowing for early management, limiting functional deterioration, and promoting satisfactory neuromusculoskeletal recovery. This case highlights the need to consider neural injuries as a possible, albeit rare, complication associated with laparoscopic procedures.
